# Polymer Composition Modulates Dental Stem Cell Response and Mineralization in Electrospun Scaffolds for Hard Tissue Regeneration

**DOI:** 10.3390/biomimetics11080518

**Published:** 2026-07-23

**Authors:** Caroline Anselmi, Sepideh Aminmansour, Igor Paulino Mendes Soares, Alexandre Henrique dos Reis-Prado, Sahar Aminmansour, Owen Liepman, Renan Dal-Fabbro, Josimeri Hebling, Marco C. Bottino

**Affiliations:** 1 Department of Cariology, Restorative Sciences, and Endodontics, School of Dentistry, University of Michigan, Ann Arbor, MI 48109, USA; caroline.anselmi-oliveira@unesp.br (C.A.); saminmansour@ufl.edu (S.A.); igor.soares@unesp.br (I.P.M.S.); alexandreprado.cba@gmail.com (A.H.d.R.-P.); amsahar@umich.edu (S.A.); oliepman@umich.edu (O.L.); renandf@umich.edu (R.D.-F.); josimeri.hebling@unesp.br (J.H.); 2 Department of Oral and Maxillofacial Diagnostic Sciences, College of Dentistry, University of Florida, Gainesville, FL 32610, USA; 3 Department of Dental Materials and Prosthodontics, School of Dentistry, São Paulo State University (UNESP), Araraquara 14801-385, SP, Brazil; 4 Department of Restorative Dentistry, School of Dentistry, Minas Gerais Federal University (UFMG), Belo Horizonte 31270-901, MG, Brazil; 5 Department of Morphology and Pediatric Dentistry, School of Dentistry, São Paulo State University (UNESP), Araraquara 14801-385, SP, Brazil; 6 Department of Biomedical Engineering, College of Engineering, University of Michigan, Ann Arbor, MI 48109, USA

**Keywords:** polycaprolactone, polydioxanone, gelatin methacryloyl, dental pulp, periodontal ligament, bone regeneration

## Abstract

Material selection is crucial to hard tissue regeneration, and matching scaffold properties to those of the target tissue can improve clinical outcomes. This study compared the physicochemical, mechanical, and biological performance of fibrous scaffolds fabricated from polycaprolactone (PCL), polydioxanone (PDO), and gelatin methacryloyl (GelMA) for hard tissue regeneration. Polymeric fibers were produced by electrospinning, and their morphological, physical, and mechanical properties were characterized by scanning electron microscopy (SEM, *n* = 2), swelling and degradation analyses (*n* = 8), water contact angle measurements (*n* = 16), and tensile testing (*n* = 8). In addition, periodontal ligament stem cells (PDLSCs), alveolar bone marrow stem cells (aBMSCs), and dental pulp stem cells (DPSCs) were seeded onto the scaffolds to evaluate cell spreading (*n* = 4), viability (*n* = 8), and mineralized matrix formation (*n* = 6). Data were analyzed using one- or two-way ANOVA followed by appropriate post hoc tests (α = 5%). All polymers formed homogeneous fibrous scaffolds, with diameters within the nanoscale range. PDO and GelMA showed higher swelling than PCL, while PCL retained approximately 95% of its initial mass after three months. PCL and PDO showed higher elongation at break, tensile strength, and Young’s modulus than GelMA. Both PDO and GelMA displayed contact angles below 90°, with GelMA showing the lowest values. In vitro, all polymers were cytocompatible: PDO and GelMA enhanced DPSC viability at 7 days, whereas GelMA produced the highest viability for PDLSCs and aBMSCs at that time point. GelMA also promoted the highest mineralized matrix formation for DPSCs and PDLSCs, with no significant differences among polymers for aBMSCs. Overall, GelMA scaffolds promoted greater cell viability and mineralized matrix formation, while PCL and PDO provided superior mechanical properties, highlighting the importance of balancing biological and mechanical requirements when designing scaffolds for hard tissue regeneration.

## 1. Introduction

Tooth loss is a major epidemiological indicator of oral health status within populations [1]. Its primary etiological factors include dental trauma, caries, and periodontal disease; the latter two affect nearly half of the global population and represent major chronic public health concerns [2,3]. Although conventional therapies can replace lost tissues, they are often limited by restricted longevity and may ultimately require material replacement. In this context, tissue engineering has emerged as a promising long-term strategy for regenerating mineralized dental tissues, including dentin, cementum, and alveolar bone, through scaffolds that act as delivery platforms for bioactive molecules, drugs, and stem cells [4,5].

The choice of an appropriate biomaterial is determined primarily by the structural and functional characteristics of the target tissue [6]. Indeed, a central challenge in tissue engineering is to design scaffolds that replicate native tissue architecture while preserving optimal biological functionality. In regenerative dentistry, both natural and synthetic polymeric scaffolds have been investigated for tissue-specific regeneration [7,8,9,10]. Even so, scaffold design for dental and bone regeneration remains challenging due to the complexity of cell–biomaterial interactions, particularly those involving mesenchymal stem cells (MSCs), a cell population that holds great promise for tissue regeneration owing to its accessibility, multipotency, and self-renewal capacity [11,12].

Among the various MSC sources, periodontal ligament stem cells (PDLSCs) possess strong immunomodulatory and regenerative capabilities, positioning them as promising candidates for regenerating periodontal supporting tissues [13]. Likewise, alveolar bone-derived MSCs (aBMSCs) are crucial for bone regeneration, exhibiting higher mineralization and osteogenic gene activity compared to other bone marrow-derived MSCs [14]. For pulp-dentin complex regeneration, dental pulp stem cells (DPSCs) have shown strong potential for odontogenic and neurogenic differentiation [12,15]. Because each of these populations responds differently to its environment, understanding how scaffold properties modulate cellular behavior is essential for achieving predictable regenerative outcomes. Therefore, balancing degradation rate, mechanical stability, and stem cell cytocompatibility remains a major challenge in scaffold design. Both the chemical composition and the physical properties of biomaterial surfaces, including porosity, mechanical strength, degradation rate, and stiffness, are essential in regulating cell adhesion, proliferation, and differentiation [6,16,17,18].

Among the polymers commonly used for scaffold fabrication, polycaprolactone (PCL), polydioxanone (PDO), and gelatin methacryloyl (GelMA) offer complementary physicochemical and biological characteristics well suited to tissue engineering. PCL is a synthetic, biodegradable, petroleum-derived polyester characterized by high mechanical strength, slow degradation, and favorable processability, which make it advantageous for load-bearing scaffolds [19,20,21]. PDO is a resorbable polyester whose degradation occurs primarily through hydrolytic cleavage of ester bonds, producing an intermediate degradation profile compared with slowly degrading PCL and rapidly degrading GelMA-based scaffolds [19]. GelMA, by contrast, is a naturally derived polymer with promising photo-crosslinking properties and a structure resembling the native extracellular matrix, which together promote high biocompatibility, cell adhesion, and enzymatic degradability [22,23]. Its relatively low mechanical strength, however, limits its use in bone tissue engineering [22,24]. These three polymers were selected because they represent complementary scaffold categories relevant to hard tissue regeneration: PCL provides long-term mechanical stability, PDO offers an intermediate resorption profile with established surgical use, and GelMA provides an extracellular matrix-mimetic platform with high cell-interactive potential. Understanding the properties of these polymers and their interactions with MSCs is thus fundamental to designing scaffolds that effectively promote stem cell adhesion, proliferation, and lineage-specific differentiation and ultimately support mineralized tissue regeneration.

To the best of our knowledge, no laboratory-based study has systematically compared the performance of multiple polymers in interaction with different stem cell types relevant to mineralized dental tissue regeneration under standardized conditions. Accordingly, this study investigated the relationship between polymer type and stem cell behavior to provide insights to guide the rational design of biomaterials for dental tissue engineering. The null hypothesis was that polymer type does not significantly influence the cytocompatibility or mineralized matrix formation of DPSCs, PDLSCs, and aBMSCs, implying that polymer selection may be guided primarily by the mechanical and structural requirements of complex tissue regeneration.

## 2. Materials and Methods

### 2.1. Scaffold Fabrication via Electrospinning

Scaffolds were fabricated via electrospinning of three different polymeric solutions, with parameters optimized for each polymer. For the PCL group, polycaprolactone (PCL; Mw = 50,000 g/mol; Cellink, Boston, MA, USA) was dissolved at 10% *w*/*v* in 1,1,1,3,3,3-hexafluoroisopropyl alcohol (HFIP; Chem-Impex, Wood Dale, IL, USA) under stirring for 24 h at room temperature, then electrospun through a 25 G needle at a flow rate of 1 mL/h and a voltage of 15 kV, with a collector distance of 20 cm and collector rotation of 120 rpm. For the PDO group, polydioxanone (PDO; Resomer X 206 S; Mw = 102,088 g/mol; St. Louis, Sigma-Aldrich, MO, USA) was dissolved at 7.5% *w*/*v* in HFIP (Chem-Impex) under stirring for 24 h at room temperature, then electrospun through a 27G needle at a flow rate of 1 mL/h and a voltage of 20 kV, with a collector distance of 20 cm and collector rotation of 120 rpm. For the GelMA group, GelMA, produced from Type-A gelatin and methacrylic anhydride (Sigma-Aldrich), was dissolved at 15% *w*/*v* in glacial acetic acid (ACS grade; Fisher Scientific, Pittsburgh, PA, USA) under stirring for 24 h at 60 °C, then electrospun through a 27G needle at a flow rate of 1.8 mL/h and a voltage of 18–20 kV, with a collector distance of 15 cm and collector rotation of 160 rpm. Electrospinning parameters, including needle gauge, were optimized for each polymer solution to obtain continuous and bead-free fibers. Therefore, although the standardized testing conditions allowed direct comparison among the resulting scaffolds, differences in fiber diameter and mechanical behavior may reflect both polymer composition and polymer-specific processing requirements.

To ensure GelMA stability in aqueous environments, a crosslinking step was performed. Lithium phenyl-2,4,6-trimethylbenzoylphosphinate (LAP, 0.075% *w*/*v*; Sigma-Aldrich) was used as the photoinitiator and added to the polymer solution 1 h before electrospinning, and the solution was protected from light with aluminum foil. After electrospinning, the fibrous meshes were placed in a vacuum oven for at least 24 h to allow complete solvent evaporation. The scaffolds were then cut and photocrosslinked. An 85% (*v*/*v*) isopropyl alcohol solution was applied as a wetting agent, and the samples were photocured in a 405 nm LED curing chamber for 3 min on each side, according to previously established and validated protocols for GelMA-based fibrous scaffolds [8,25,26,27].

### 2.2. Morphological Characterization

The surface morphology of the scaffolds was evaluated by scanning electron microscopy (SEM; TESCAN MIRA3 FEG-SEM, Brno, Czech Republic). Two samples from each group (10 × 10 mm^2^), either crosslinked or non-crosslinked, were mounted on metallic stubs, sputter-coated with Au-Pd (80/20), and analyzed at magnifications of 2000× and 5000×. Fiber diameters were measured with ImageJ software (Version 2.14.0/1.54f, U.S. National Institutes of Health, Bethesda, MD, USA) by analyzing 50 fibers in each of three different areas per scaffold (*n* = 150).

### 2.3. Swelling and Degradation Ratios

The swelling and degradation ratios were assessed using 10 × 10 mm samples. The fibrous meshes were cut, crosslinked, and weighed, and samples of similar weight (20 ± 3 mg) were selected for analysis. For the swelling assessment, the initial dry weight (W_0_) of each sample was measured after complete evaporation of the solvent. Samples were then immersed in PBS and incubated at 37 °C. After 24 h, they were removed, gently blotted with low-lint paper to remove excess surface liquid, and weighed (W_t_). The swelling capacity was calculated as: Swelling (%) = (W_t_/W_0_) × 100. For the degradation analysis, samples were first weighed (W_0_) and then immersed in 5 mL of PBS for up to three months. At predetermined time points (1, 7, and 14 days, and 1, 2, and 3 months), the samples were collected, washed twice with distilled water, and dried under vacuum at room temperature for 24 h before the final dry weight (W_d_) was recorded. The degradation ratio was expressed as the remaining mass (%) using the formula: Remaining mass (%) = (W_d_/W_0_) × 100.

### 2.4. Mechanical Characterization

Uniaxial tensile testing (Expert 5601, ADMET, Inc., Norwood, MA, USA) was performed to evaluate the mechanical properties of the scaffolds. Eight specimens (15 mm × 3 mm) were tested per group. Before tensile testing, samples were immersed in PBS for 2 h at 37 °C to evaluate mechanical behavior under hydrated conditions, which better approximates the aqueous environment expected during biological use. Since hydration may affect synthetic and GelMA-based fibers differently, this condition was maintained for all groups and is considered when interpreting polymer-dependent differences in mechanical performance. The tests were then conducted at a crosshead speed of 1 mm/min. Sample thickness was measured with a digital caliper (Mitutoyo Corp., Kanagawa, Japan) at three different points on each specimen. The mechanical properties were reported as tensile strength (MPa), Young’s modulus (MPa), and elongation at break (%).

### 2.5. Wettability

Surface wettability was evaluated by contact angle measurements using a goniometer (SCA20, DataPhysics Instruments GmbH, Filderstadt, BW, Germany). Square samples (10 × 10 mm) were mounted on glass slides with double-sided adhesive tape, ensuring a smooth, flat surface free of folds or irregularities. A 1 μL droplet of deionized water was deposited onto the dry fibrous surface of each sample. After a 2 s stabilization period, an image of the droplet was recorded, and the contact angle formed at the liquid-scaffold interface was determined using the instrument’s software (DROPimage Standard, Netcong, NJ, USA).

### 2.6. Scaffolds-Stem Cells Interaction

The scaffolds were tested in contact with three different stem cell types to simulate distinct dental regenerative applications, with each cell type cultured according to its specific requirements. Dental pulp stem cells (DPSCs; Lonza, #PT-5025, Walkersville, MD, USA) were cultured in α-MEM (+L-glutamine, −ribonucleosides, −deoxyribonucleosides; Gibco, Invitrogen, Carlsbad, CA, USA; #12561056) supplemented with 15% fetal bovine serum (FBS; Gibco, #16000044) and 1% penicillin/streptomycin (Gibco, #16000044), and used at passages #4–7. Periodontal ligament stem cells (PDLSC), obtained from primary culture isolated as previously described [9], were cultured in α-MEM (+L-glutamine, −ribonucleosides, −deoxyribonucleosides; Gibco, #12561056) supplemented with 10% FBS (Gibco, #16000044) and 1% penicillin/streptomycin (Gibco, #16000044), and used at passages #4–6. Alveolar bone mesenchymal stem cells (aBMSCs), obtained from primary culture isolated as previously described [25], were cultured in α-MEM (+L-glutamine, +ribonucleosides, +deoxyribonucleosides; Gibco, #12571063) supplemented with 15% FBS (Gibco, #16000044) and 1% penicillin/streptomycin (Gibco, #16000044), and used at passages #5–7.

#### 2.6.1. Cell Spreading, Adhesion, Viability, and Proliferation

Cells of each type were seeded onto the scaffolds and maintained in complete culture medium for up to 7 days. For morphological assessment, samples were collected at 1, 3, and 7 days, fixed with 4% buffered paraformaldehyde, and stained to visualize cytoskeletal organization and nuclei. Actin filaments were labeled with a green fluorescent probe (ActinGreen 488 ReadyProbes; 1:20 in PBS; Invitrogen), and nuclei were counterstained with DAPI (1:5000 in PBS; Invitrogen). Fluorescence images were acquired with a microscope (Echo Revolve, BICO Company, San Diego, CA, USA) to qualitatively evaluate cell adhesion and spreading on the scaffold surfaces.

Cell metabolic activity was assessed using the alamarBlue (Invitrogen) assay at 1, 3, and 7 days. At each time point, samples were incubated with a 10% alamarBlue solution prepared in FBS-free medium for 3 h at 37 °C. The supernatant was then collected, and fluorescence intensity was measured at an excitation wavelength of 555 nm and an emission wavelength of 595 nm using a microplate reader (SpectraMax iD3, Molecular Devices LLC, San Jose, CA, USA). Cell metabolic activity was assessed using alamarBlue and interpreted as a relative indicator of cell metabolism over time. The same number of cells was seeded onto all scaffolds, and metabolic activity was evaluated after 24 h, 3 days, and 7 days. Data were normalized to the PCL group at day 1, which was set as 100%, because PCL is a well-established reference polymer in electrospun scaffold studies [28,29]. This normalization strategy allowed comparison of material-dependent changes using a common reference group across all experimental periods.

#### 2.6.2. Mineralized Matrix Formation

Mineralized matrix formation was evaluated by culturing cells under osteo/odontogenic conditions. Twenty-four hours after seeding, the culture medium was replaced with differentiation medium consisting of complete medium supplemented with 0.1 μM dexamethasone, 10 mM β-glycerophosphate, and 50 μg/mL ascorbic acid. The medium was renewed every two days, and the samples were maintained for up to 21 days.

At 14 and 21 days, the cell-scaffold constructs were fixed in 70% ethanol and stained with 40 mM Alizarin Red S solution (pH 4.2; Sigma-Aldrich) to detect calcium deposits. Excess dye was removed by repeated washing with deionized water, and mineralized deposits were visualized under a stereomicroscope (ZEISS Stemi 508; Zeiss, Oberkochen, Germany). Stereomicroscope images were acquired from representative central areas of the scaffolds using standardized imaging conditions. For quantitative analysis, the bound stain was extracted with 10 mM cetylpyridinium chloride solution (pH 7.0; Sigma-Aldrich) for 20 min, and the absorbance was measured at 570 nm using a microplate reader (Synergy H1). Data were normalized to the control group (PCL) at 14 days, which was defined as 100% mineralized matrix formation.

### 2.7. Statistical Analysis

Statistical analyses were performed in GraphPad Prism 11 (GraphPad Software, Inc., San Diego, CA, USA) with a significance level of 5%. The sample size (*n*) varied according to data variability to ensure adequate statistical power. Sample sizes were defined based on previous comparable in vitro studies and assay-specific feasibility. For the main quantitative outcomes, achieved power was verified using the final sample size and observed effect sizes, considering α = 0.05, and was greater than 80% for the main comparisons. Data normality and homogeneity were assessed using the Shapiro–Wilk test and appropriate variance tests. Fiber diameter data were non-normally distributed and were analyzed using the Mann–Whitney test with Dunn’s post hoc test. Parametric data (swelling, mechanical properties, and contact angle) were analyzed by one-way ANOVA followed by Tukey’s post hoc test. Degradation data were analyzed using mean values and 95% confidence intervals to describe the magnitude and precision of scaffold mass loss over time. This estimation-based approach was selected to allow direct comparison of degradation profiles in the graph, avoiding excessive multiple pairwise comparisons across groups and time points. Cell viability was analyzed by repeated-measures ANOVA with Sidak’s post hoc test, mineralized matrix production was analyzed by two-way ANOVA with Sidak’s post hoc test, and degradation data were evaluated using 95% confidence intervals. Cell adhesion and spreading were assessed qualitatively.

## 3. Results

### 3.1. Morphological, Physical, and Mechanical Characterization

**Figure 1:**
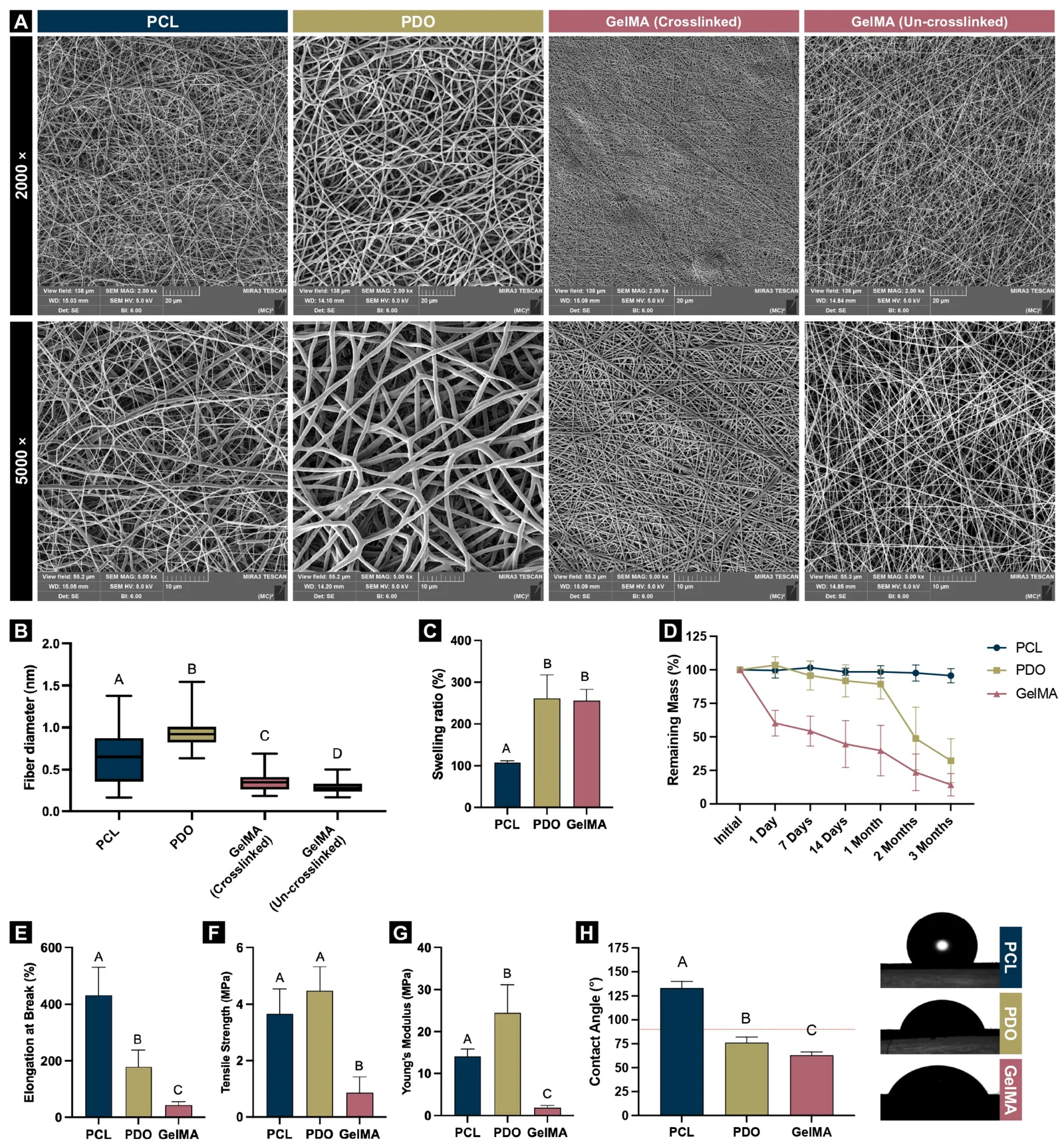
Morphological, physical, and mechanical characterization. (**A**) Scanning electron microscopy images of the polymeric fibers (2k and 5k). (**B**) Boxplots of fiber diameter (median with 25th–75th percentile range). Different letters indicate statistical differences between groups (Mann–Whitney test/Dunn’s post hoc; *n* = 150; α = 5%). (**C**) Swelling ratio of the polymeric fibers after immersion in phosphate-buffered saline (PBS) for 24 h. Mean and standard deviation (*n* = 8); different letters indicate statistical differences between groups (one-way ANOVA/Tukey; α = 5%). (**D**) Degradation profile in PBS at different time points. Symbols are means, and error bars represent 95% confidence intervals (*n* = 8). (**E**) Elongation at break (%), (**F**) tensile strength (MPa), and (**G**) Young’s modulus of the polymeric fibers after 2 h of immersion in PBS. Mean and standard deviation (*n* = 8); different letters indicate statistical differences between groups (one-way ANOVA/Tukey; α = 5%). (**H**) Contact angle between the fiber surface and a water droplet (1 μL). The horizontal red dashed line indicates the 90° limiting contact angle between the hydrophobic and hydrophilic properties. Mean and standard deviation; different letters indicate statistical differences between groups (ANOVA/Tukey; α = 5%; *n* = 16), followed by representative images of the droplet on the fiber surface.

Scanning electron microscopy revealed randomly oriented fibrous networks for all polymeric scaffolds, with smooth, continuous fibers visible at both 2k and 5k magnifications (Figure 1A). Quantitative analysis showed that the average fiber diameter differed significantly among the groups (*p* ≥ 0.0023), with PDO presenting the highest values and GelMA the lowest (Figure 1B). All formulations produced fibers with mean diameters between 200 and 600 nm, confirming a nanoscale morphology across groups. The swelling ratio was markedly lower for PCL scaffolds than for PDO and GelMA (*p* < 0.0001; Figure 1C). Consistent with these differences, the remaining mass analysis showed that PCL preserved its structural integrity over time, retaining approximately 95% of its initial mass after 3 months, whereas PDO and GelMA underwent progressive degradation, with GelMA showing the most pronounced mass loss, retaining only about 14% of its original mass.

Mechanical characterization revealed distinct profiles among the scaffolds (Figure 1E–H). For elongation at break, PCL showed the greatest deformation before failure and GelMA the lowest, with PDO intermediate (*p* ≤ 0.0014; Figure 1E). PCL and PDO fibers exhibited similar tensile strength (*p* = 0.1140; ≈4 MPa), whereas GelMA fibers showed the lowest values (*p* < 0.0001; ≈1 MPa; Figure 1F). PDO presented the highest Young’s modulus and GelMA the lowest (*p* ≤ 0.0001; Figure 1G). Wettability analysis indicated that PCL surfaces had the highest contact angle (≈133°); although PDO and GelMA differed significantly from each other (*p* < 0.0001), both showed contact angles below 90° (red dashed line), the threshold between hydrophilic and hydrophobic behavior, consistent with the representative droplet images (Figure 1H).

### 3.2. Cytocompatibility and Mineralization Potential of DPSCs

**Figure 2:**
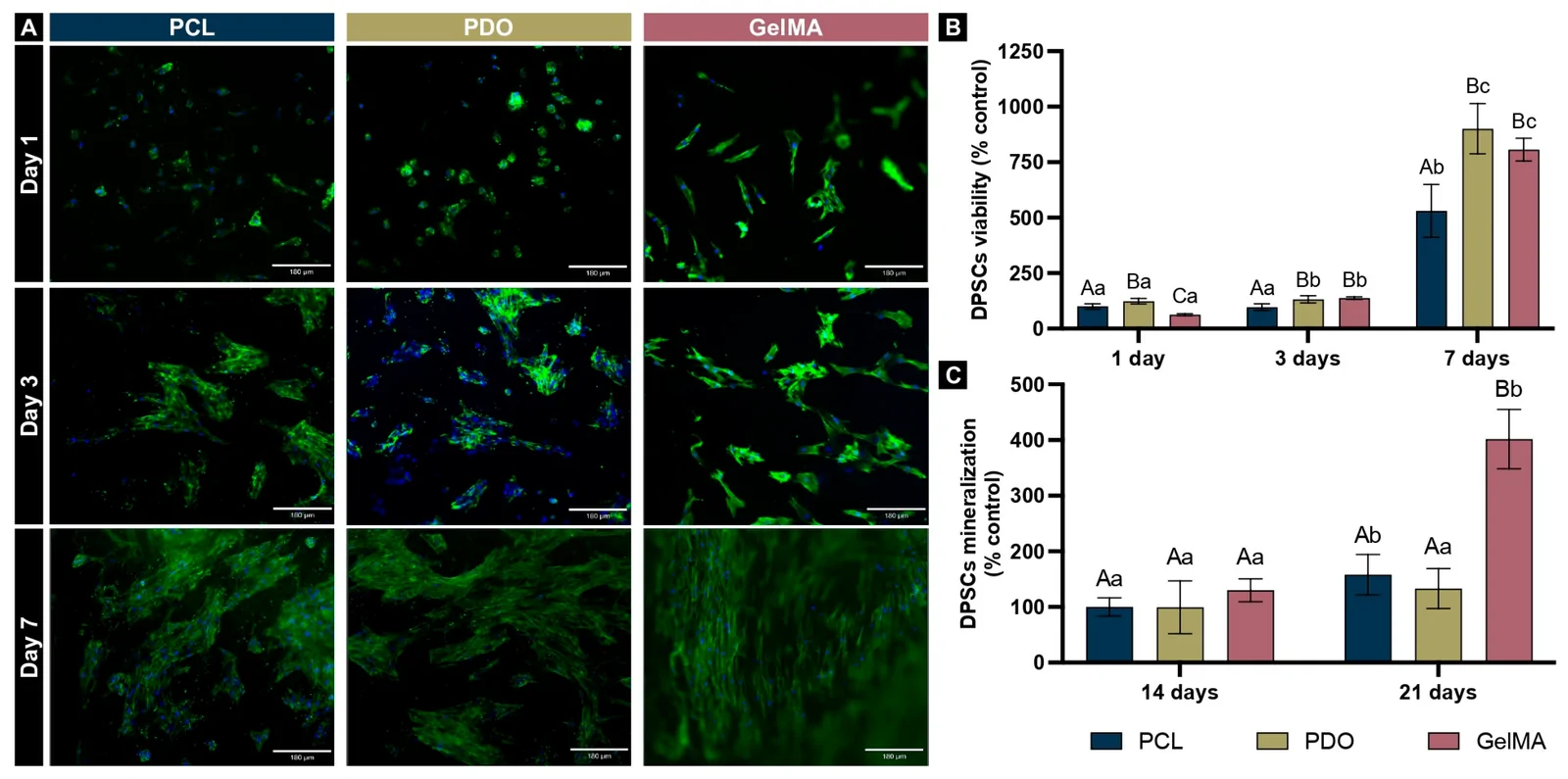
Cytocompatibility and mineralization potential of DPSCs. (**A**) Adhesion and spreading of DPSCs seeded on the polymeric fibers 1, 3, and 7 days (green fluorescence: actin filaments; blue fluorescence: cell nuclei). (**B**) Viability of DPSCs seeded on the polymeric fibers 1, 3, and 7 days. Different capital letters indicate significant differences between groups; different lowercase letters indicate significant differences between time points (repeated-measures ANOVA/Sidak post hoc; α = 5%; *n* = 8). (**C**) Mineralized matrix production by DPSCs seeded on the polymeric fibers after 14 and 21 days. Different capital letters indicate significant differences between groups; different lowercase letters indicate significant differences between time points (two-way ANOVA/Sidak post hoc; α = 5%; *n* = 6).

Fluorescence microscopy showed that DPSCs adhered and spread on all polymeric fibers, with progressive proliferation and network formation from day 1 to day 7 (Figure 2A). Cells displayed an elongated morphology on PCL and PDO fibers, whereas a denser cell layer formed on GelMA at later time points. Quantitatively, cell viability increased over time in all groups, confirming the cytocompatibility of every formulation (Figure 2B); PDO and GelMA supported higher viability than PCL on days 3 and 7 (*p* ≤ 0.005). Mineralized matrix production likewise increased over time for PCL and GelMA (*p* ≤ 0.0341), and at 21 days, GelMA fibers showed the highest mineral deposition (*p* < 0.0001), while PCL and PDO were comparable (*p* = 0.4873; Figure 2C).

### 3.3. Cytocompatibility and Mineralization Potential of PDLSCs

**Figure 3:**
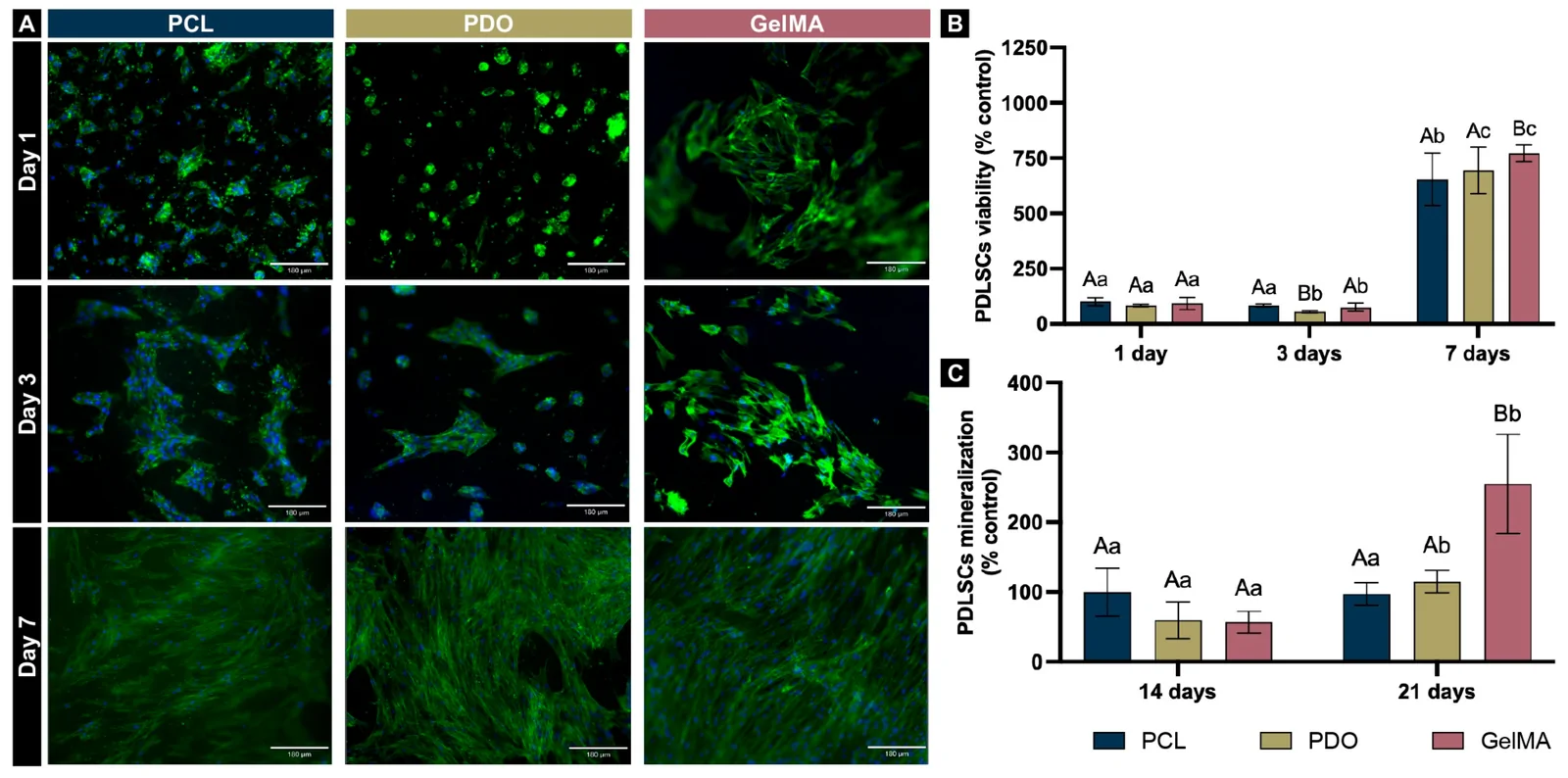
Cytocompatibility and mineralization potential of PDLSCs. (**A**) Adhesion and spreading of PDLSCs seeded on the polymeric fibers 1, 3, and 7 days (green fluorescence: actin filaments; blue fluorescence: cell nuclei). (**B**) Viability of PDLSCs seeded on the polymeric fibers 1, 3, and 7 days. Different capital letters indicate significant differences between groups; different lowercase letters indicate significant differences between time points (repeated-measures ANOVA/Sidak post hoc; α = 5%; *n* = 8). (**C**) Mineralized matrix production by PDLSCs seeded on the polymeric fibers after 14 and 21 days. Different capital letters indicate significant differences between groups; different lowercase letters indicate significant differences between time points (two-way ANOVA/Sidak post hoc; α = 5%; *n* = 6).

All polymeric fibers supported PDLSC adhesion and spreading over time, with increasing cell density and cytoskeletal organization from day 1 to day 7 (Figure 3A). Cell viability rose significantly in all groups by day 7 (*p* ≤ 0.022), and GelMA produced the highest viability compared with PCL and PDO at that time point (*p* ≤ 0.014; Figure 3B). Mineralized matrix production increased from day 14 to day 21 for PDO and GelMA (*p* ≤ 0.0356), with GelMA again showing the greatest enhancement at the latest time point (*p* < 0.0001; Figure 3C).

### 3.4. Cytocompatibility and Mineralization Potential of aBMSCs

**Figure 4:**
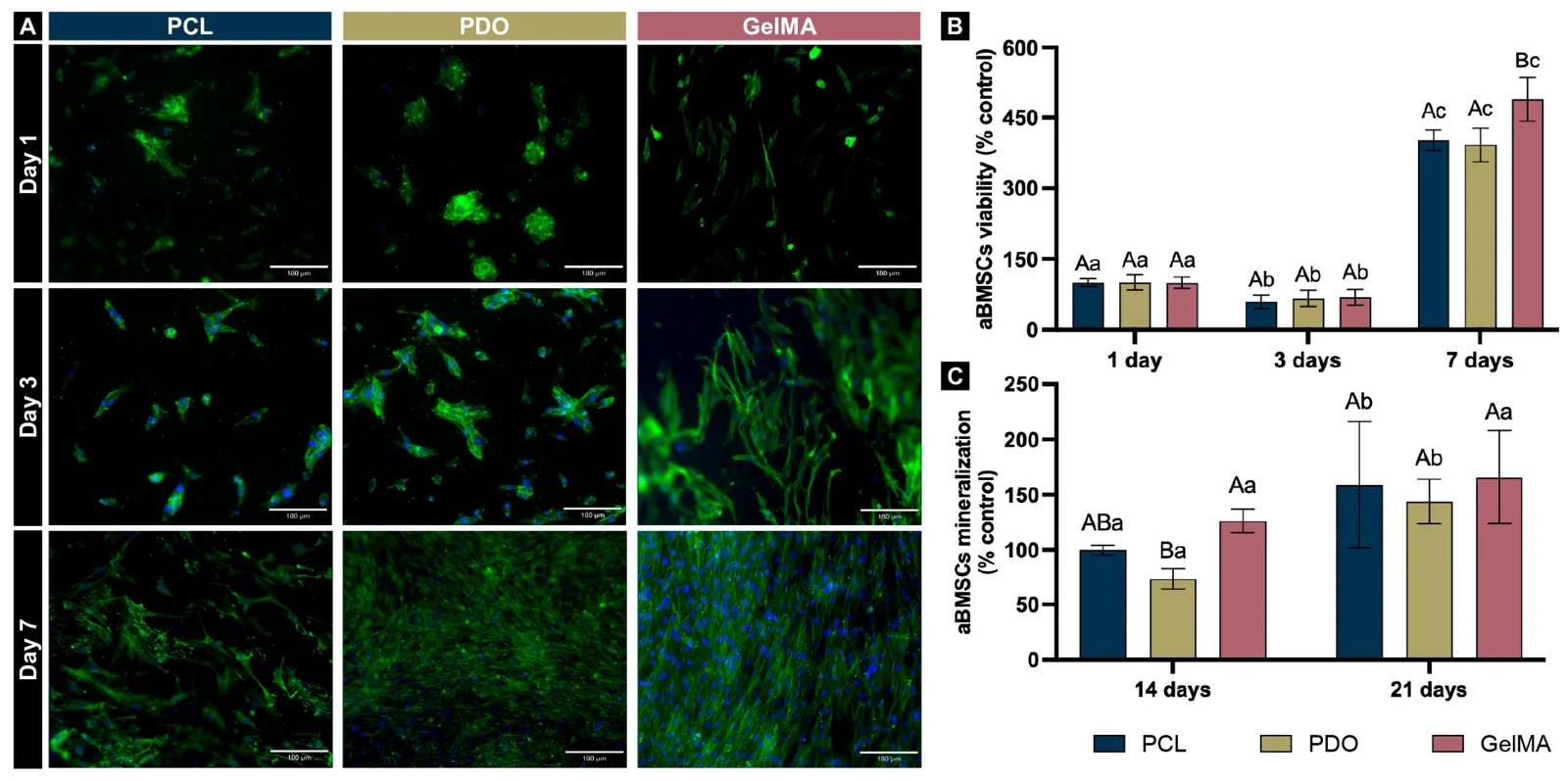
Cytocompatibility and mineralization potential of aBMSCs. (**A**) Adhesion and spreading of aBMSCs seeded on the polymeric fibers after 1, 3, and 7 days (green fluorescence: actin filaments; blue fluorescence: cell nuclei). (**B**) Viability of aBMSCs seeded on the polymeric fibers 1, 3, and 7 days. Different capital letters indicate significant differences between groups; different lowercase letters indicate significant differences between time points (repeated-measures ANOVA/Sidak post hoc; α = 5%; *n* = 8). (**C**) Mineralized matrix production by aBMSCs seeded on the polymeric fibers after 14 and 21 days. Different capital letters indicate significant differences between groups; different lowercase letters indicate significant differences between time points (two-way ANOVA/Sidak post hoc; α = 5%; *n* = 6).

Fluorescence imaging revealed progressive adhesion and spreading of aBMSCs over time, with greater cell density and cytoskeletal organization by day 7 on all polymeric materials (Figure 4A). Cell viability increased in a time-dependent manner (*p* ≤ 0.013), with significantly higher proliferation on GelMA than on PCL and PDO after 7 days (*p* ≤ 0.032; Figure 4B). For mineralized matrix formation, deposition increased from day 14 to day 21 across all scaffolds, with no significant differences among materials at the final time point (Figure 4C). Thus, although all scaffolds supported aBMSC adhesion, proliferation, and differentiation, GelMA provided a slightly more favorable microenvironment for cell growth, in agreement with the results obtained for DPSCs and PDLSCs.

## 4. Discussion

Regeneration of tooth-supporting hard tissues using polymeric fabrication strategies combined with stem cell-based approaches has attracted increasing attention, particularly to preserve the vitality of the remaining pulp tissue and ensure favorable long-term clinical outcomes. The present study evaluated the effects of three polymeric fibers (PCL, PDO, and GelMA) on stem cell viability and mineralized matrix formation. GelMA, valued for its ability to mimic the extracellular matrix (ECM) and to provide favorable properties for engineering bioactive and responsive scaffolds [25,30,31], showed enhanced biological performance, maintaining stem cell viability and promoting greater mineralized matrix formation than the synthetic polymers tested. The null hypothesis was therefore rejected. Notably, each polymer elicited a distinct cellular response, likely reflecting differences in chemical composition, degradation profile, and mechanical behavior.

In regenerative dentistry, fiber-based scaffolds have gained considerable attention, particularly for wound healing and drug delivery [8,25,32]. Here, electrospun synthetic and natural fibers were produced and first characterized for diameter and mechanical behavior. All scaffolds exhibited a uniform nanoscale morphology, consistent with previous reports that fiber diameters between 200 and 600 nm favor MSC adhesion, spreading, and cytoskeletal organization [6,8].

Surface wettability was assessed by water contact angle measurements, and all fiber types fell within a range generally considered favorable for cell adhesion. The more hydrophilic surfaces of PDO and GelMA, reflected in contact angles below 90°, may have promoted protein adsorption and integrin-mediated cell attachment [33,34]. The substantially larger fiber diameter of PDO scaffolds may, in turn, reflect differences in solution viscosity or charge density during electrospinning [35] and may have contributed to the enhanced DPSC metabolic activity and proliferation observed on PDO at later time points. Differences in hydrophilicity and degradation kinetics further shaped the biological responses. PCL’s relatively slow degradation and hydrophobic nature are limitations, as they may impair cell adhesion and proliferation [36,37], making PCL less suitable for short-term delivery of drugs and bioactive molecules. Although PCL supported MSC adhesion and viability in this study, it consistently showed lower metabolic activity than GelMA and PDO during the early analysis periods.

Nanofibrous scaffolds can perform particularly well at certain anatomical sites, such as mineralized structures, owing to their greater mechanical stability compared with hydrogels [38]. For reference, alveolar (trabecular) bone has a Young’s modulus between 0.05 and 0.2 GPa (50–200 MPa) [39], whereas dentin reaches substantially higher values of 18 to 22 GPa. As expected, the synthetic fibers showed higher mechanical strength than the semi-natural GelMA fibers [40]. Beyond its lower mechanical performance, the rapid degradation of GelMA in dynamic environments remains a significant barrier to clinical use, especially in load-bearing applications. In the present study, GelMA promoted higher cell metabolic activity and mineralized matrix deposition, especially for DPSCs and PDLSCs, but showed lower tensile strength, lower Young’s modulus, and faster degradation than the synthetic polymers. Therefore, GelMA-based formulations may be most appropriate when high bioactivity is prioritized, whereas hybrid or reinforced formulations may be necessary when mechanical stability or slower degradation is required [41]. PDO scaffolds, by contrast, exhibited intermediate degradation and swelling behavior, which may enhance local water retention and nutrient diffusion, features particularly advantageous for highly proliferative cell types such as DPSCs.

Among the polymers tested, GelMA showed the most favorable biological performance. As a hybrid biomaterial, it combines the advantages of natural and synthetic components, offering thermosensitive gelation, tunable degradability, high biocompatibility, and a 3D architecture that supports cell growth and mineralized matrix formation [42,43]. Cell adhesion and spreading were assessed qualitatively from fluorescence images. Because electrospun scaffolds present a three-dimensional fibrous architecture, 2D measurements such as projected cell area or aspect ratio may be influenced by focal plane, scaffold thickness, cell infiltration, and fiber topography. Therefore, fluorescence imaging was used to document cell attachment and morphology, while metabolic activity and mineralized matrix formation were used as quantitative biological outcomes. Earlier work has similarly highlighted the value of GelMA hydrogels in dental tissue regeneration, showing that they effectively support DPSC attachment and proliferation [44,45]. Its ECM-mimetic structure and enzymatic degradability create a microenvironment conducive to MSC proliferation and osteogenic/odontogenic differentiation [42]. In the present study, GelMA scaffolds consistently promoted greater cell viability at later time points across all cell types and produced the highest mineralized matrix deposition, particularly at 21 days. Consistent with these findings, independent studies have shown that GelMA-based and GelMA-composite platforms can support osteogenic differentiation and bone regeneration, particularly when combined with reinforcing phases or bioactive components [23,24,41,43].

Meeting specific clinical requirements in tissue regeneration depends on identifying suitable materials, appropriate polymer ratios, and optimized electrospinning parameters [32]. A key strength of this study is the direct comparison of multiple polymers under standardized fabrication and testing conditions. By keeping electrospinning parameters, culture protocols, and analytical methods constant, we provide a controlled assessment of how polymer composition alone modulates stem cell response, an important step toward translating scaffold technologies into clinical oral applications. The simultaneous evaluation of clinically relevant MSC sources also offers understanding into shared cellular responses across the tissues commonly targeted in oral regenerative therapy, especially those involving mineralized tissue repair. Considering the present results, GelMA appears more suitable for biologically driven pulp-dentin and periodontal regenerative strategies where cell attachment and mineralized matrix formation are prioritized, particularly for DPSCs and PDLSCs. In contrast, PCL and PDO may be more appropriate for applications requiring greater mechanical stability or longer structural persistence. For alveolar bone-related strategies, the choice may depend on whether biological stimulation or mechanical support is the primary design objective.

Nonetheless, this study presents certain limitations. The in vitro setting does not fully recapitulate the biomechanical loading, immune signaling, and angiogenesis that occur during in vivo tissue repair, especially given the intricate structural and functional interactions within the periodontium. In addition, the biological outcomes were based primarily on metabolic activity and mineralized matrix deposition assays, which cannot fully elucidate the molecular mechanisms underlying stem cell behavior or confirm lineage-specific differentiation. The absence of inflammatory, multicellular, and dynamic microenvironmental conditions may further limit extrapolation to clinical scenarios. Future studies using 3D constructs, co-culture systems, and dynamic bioreactor-based platforms may better reproduce the physiological cues involved in periodontal and alveolar bone regeneration by delivering physiologically relevant mechanical stimuli to adherent cells. From a translational perspective, the most relevant findings are the distinct trade-offs among the tested scaffolds: GelMA favored stem cell metabolism and mineralized matrix formation, whereas PCL and PDO provided greater mechanical stability and slower degradation. Nevertheless, these findings were obtained in vitro and require validation in models that include vascularization, immune signaling, enzymatic degradation, and functional loading before clinical application can be inferred. Incorporating mineralizing agents or other bioactive molecules may also enhance scaffold performance, particularly for PCL and PDO, which could benefit from targeted functionalization, and may strengthen the anti-inflammatory properties of GelMA, a critical factor in early tissue healing and subsequent mineralized tissue deposition [25,41].

## 5. Conclusions

Within the limitations of this in vitro study, all electrospun polymeric scaffolds supported stem cell adhesion, viability, and mineralized matrix formation, demonstrating their potential for hard tissue engineering. Polymer type, however, significantly influenced the physicochemical, mechanical, and biological behavior of the scaffolds. GelMA exhibited the most favorable biological performance, promoting higher cell viability and greater mineralized matrix deposition across the cell types evaluated, whereas PCL and PDO offered superior mechanical stability and slower degradation. These findings highlight the need to balance biological and mechanical properties when choosing scaffold materials for mineralized tissue regeneration, supporting the use of polymer-specific strategies tailored to the intended clinical purpose.

## Data Availability

The original contributions outlined in this study are encompassed within the article. For additional inquiries, please direct your questions to the corresponding author.
